# Engaging patients in designing a transmural allied health pathway: A qualitative exploration of hospital‐to‐home transitions

**DOI:** 10.1111/hex.13996

**Published:** 2024-03-15

**Authors:** Juul W. M. van Grootel, Romain J. Collet, Mel E. Major, Suzanne Wiertsema, Hanneke van Dongen, Marike van der Leeden, Edwin Geleijn, Raymond Ostelo, Marike van der Schaaf

**Affiliations:** ^1^ Department of Rehabilitation Medicine, Amsterdam University Medical Centers University of Amsterdam, Amsterdam Movement Sciences Amsterdam The Netherlands; ^2^ Amsterdam Movement Sciences, Ageing and Vitality Amsterdam The Netherlands; ^3^ Amsterdam Movement Sciences, Musculoskeletal Health Amsterdam The Netherlands; ^4^ Department of Health Sciences, Faculty of Science Vrije University Amsterdam The Netherlands; ^5^ Department of Epidemiology and Biostatistics, Amsterdam UMC Vrije Universiteit Amsterdam Amsterdam The Netherlands; ^6^ Department of Physical Therapy, Faculty of Health Amsterdam University of Applied Sciences Amsterdam The Netherlands; ^7^ Department of Physical Therapy, Centre of Expertise Urban Vitality, Faculty of Health Amsterdam University of Applied Sciences Amsterdam The Netherlands; ^8^ Amsterdam Movement Sciences, Rehabilitation and Development Amsterdam The Netherlands

**Keywords:** allied health personnel, delivery of healthcare, integrated, patient discharge, primary health care, transitional care

## Abstract

**Introduction:**

The transition from hospital to home is often suboptimal, resulting in patients not receiving the necessary allied healthcare after discharge. This may, in turn, lead to delayed recovery, a higher number of readmissions, more emergency department visits and an increase in mortality and healthcare costs. This study aimed to gain insight into patients' experiences, perceptions, and needs regarding hospital‐to‐home transition, focusing on allied healthcare as a first step towards the development of a transitional integrated allied healthcare pathway for patients with complex care needs after hospital discharge.

**Methods:**

We conducted semistructured interviews with patients. Participants were recruited from universities and general hospitals in the Amsterdam region between May and July 2023. They were eligible if they (1) were discharged from the hospital minimally 3 and maximally 12 months after admission to an oncologic surgery department, internal medicine department, intensive care unit, or trauma centre, (2) received hospital‐based care from at least one allied healthcare provider, who visited the patient at least twice during hospital admission, (3) spoke Dutch or English and (4) were 18 years or older. Interviews were audio‐recorded and transcribed verbatim. We performed a thematic analysis of the interview data.

**Results:**

Nineteen patients were interviewed. Three themes emerged from the analysis. ‘Allied healthcare support during transition’ depicts patients' positive experiences when they felt supported by allied health professionals during the hospital‐to‐home transition. ‘Patient and family involvement’ illustrates how much patients value the involvement of their family members during discharge planning. ‘Information recall and processing’ portrays the challenges of understanding and remembering overwhelming amounts of information, sometimes unclear and provided at the wrong moment. Overall, patients' experiences of transitional care were positive when they were involved in the discharge process. Negative experiences occurred when their preferences for postdischarge communication were ignored.

**Conclusions:**

This study suggests that allied health professionals need to continuously collaborate and communicate with each other to provide patients and their families with the personalized support they need. To provide high‐quality and person‐centred care, it is essential to consider how, when, and what information to provide to patients and their families to allow them to contribute to their recovery actively.

**Patient or Public Contribution:**

The interview guide for this manuscript was developed with the assistance of patients, who reviewed it and provided us with feedback. Furthermore, patients provided us with their valuable lived experiences by participating in the interviews conducted for this study.

## 1 INTRODUCTION

The ageing population and the number of people living with complex care needs will increase in the coming decades, coinciding with an increased need for comprehensive healthcare, including allied healthcare, across multiple care settings.^1^ Patients with complex care needs are defined as patients who suffer from multiple chronic conditions, mental health problems, drug interactions, and social vulnerability, which can lead to overuse, underuse, or misuse of healthcare services.^2^ In 2018, 27.2% of US adults had multiple chronic conditions.^3^ In The Netherlands, in 2021, 5.7 million people suffer from more than one chronic condition.^4^ Due to the growing population with complex care needs, the stress on the healthcare sector will likely increase since these patients require more attention as their treatments are complex.^5^ In addition, healthcare costs in The Netherlands increased in 2021 by 7.6% to 125 billion euros compared to 2020.^6^ In The Netherlands, people pay fees for their health insurance. Healthcare costs are partially paid for by the state (see Appendix S1).

The hospital‐to‐home transition is a sensitive moment for patients with complex care needs. After hospitalization, there is an increased risk of adverse events, which may require medical attention and may ultimately lead to hospital readmissions.^7^ Current readmission rates range from 10% to 30%, and the associated expenses increase in equal measure.^8^ Research also shows that a significant proportion of patients with complex care needs reports a suboptimal transition from hospital to home.^9^ Ineffective communication and transfer of information during this phase can negatively affect patient safety and continuity of care.^10^, ^11^ Although previous research emphasised that a multidisciplinary approach is essential to optimise patient recovery, one‐way handovers from hospital to primary care (allied) health professionals remain the most common way of discharge communication.^12^ Allied health professionals are pivotal in ensuring optimal patient recovery. They support patients in reaching their rehabilitation goals during and after hospitalization regarding physical and cognitive functioning, psychosocial health and postdischarge needs.^13^

Previous research has focused on physician‐ and nurse‐led interventions to improve transitions regarding (nursing) care needs or medication adherence.^14^, ^15^ Meanwhile, this research study shows that access to allied healthcare during and/or after discharge can prevent or reduce the risk of readmissions.^16^, ^17^, ^18^ However, there is a need for more holistic transitional care interventions (i.e., actions designed to ensure coordination and continuity of care between different locations or levels of care) and integrated care provided (i.e. actions ensuring individuals receive the right care, in the right place, at the right time) by allied health professionals.^19^ This study aimed to gain insight into patients' experiences, perceptions and needs regarding hospital‐to‐home transition, focusing on allied healthcare as a first step towards the development of a transitional integrated allied healthcare pathway for patients with complex care needs after hospital discharge.

## 2 MATERIALS AND METHODS

This study is part of the Transmural Allied Health Pathway (TULIP)‐project, which aims to improve the transition from hospital to home for patients with complex care needs, focusing on allied healthcare. A qualitative study was conducted by the Amsterdam University Medical Center (AUMC) to obtain data on experiences, perceptions and needs surrounding the transition homewards and continuity of care. The consolidated criteria for reporting qualitative research checklist was used.^20^ The AUMC provided a waiver for this study (METC 2023.0119). Written informed consent was obtained from all participants.

Patients who had been admitted to the intensive care unit (ICU), trauma surgery, internal medicine and/or oncologic surgery wards participated in this study. Patients were eligible if they (1) had been discharged from the hospital minimally 3 and maximally 12 months before the interview, (2) received care from at least one allied health professional (physical therapist, dietician, occupational therapist or speech‐ and language therapist), who visited the patient at least twice while being in hospital, (3) spoke Dutch or English and (4) were 18 years or older. Relatives were allowed to join the interview at the patients' request.

Participants were sampled purposively on the basis of the ward they were admitted to. Patients and relatives were recruited via the aforementioned hospital wards, the AUMC outpatient clinics and patient organizations (Sepsis & Daarna, FCIC and IC Connect). The physicians of AUMC and patient organizations contributed to the recruitment of the patients. Physicians assisted in identifying eligible patients and asked patients if a researcher could contact them by phone. The primary researcher assessed the eligibility of the patients in concordance with the rules and regulations of the Medical Ethics Committee. Data collection occurred concurrently. Patient inclusion continued until no new themes emerged from the data. This point was determined together with R. J. C. and M. E. M. after transcribing, coding and analysing the first 15 interviews.

Data collection took place between May and July 2023. Interviews were held face‐to‐face at the participants' location of choice or via video call. Semistructured interviews were conducted by J. W. M. v. G. The research team members had a background in Clinical Epidemiology (R. J. C.), Human Movement Sciences (J. M. v. D./S. W.), Physiotherapy (M. v. d. S./J. W. M. v. G./R. J. C./S. W./E. G./M. v. d. L./M. E. M.) and Health Technology Assessment (J. M. v. D.). Through several meetings with experts, an interview guide was developed. The interview guide was pilot‐tested twice with patients, and questions were reformulated based on provided patient feedback. Involving patients in the development of the interview guide gave us valuable information to optimise the interview guide. The results of this pilot are not included in the analysis. See Appendix S2 for the interview guide.

In addition to the interview data, the following participant characteristics were collected: age, gender, hospital department, admission type (acute/elective), hospital (academic/general), clinical frailty score (CFS) at hospital discharge, hospital length of stay (LOS), ICU LOS, time since hospital discharge and discharge destination (home or (geriatric)rehabilitation facility). The CFS is a well‐validated scale used to quantify the degree of disability from frailty.^21^ The CFS was collapsed into three categories: nonfrail (1‐4), mild‐to‐moderately frail (5‐6) and severely frail (7‐9).^21^ The researcher determined the patients' scores based on information provided by the patient.

All interviews were audio‐recorded and transcribed verbatim. MAXQDA 2022 (VERBI Software, 2021) was used for data analysis. Data were analyzed using thematic analysis, an iterative process of reflexivity in which the researcher's subjective experience is at the centre of making sense of the data.^22^ It contained the following steps:

1. *Familiarization with the data*: transcripts were thoroughly read and reread to gain a general understanding of the participants' answers (J. W. M. v. G., M. E. M.).
2. *Generation of initial codes*: meaningful sentences or issues raised by participants were given a general name (J. W. M. v. G., M. E. M., R. J. C.).
3. *Searching for, defining, and naming theme*: the list of derived codes out of step 2 was read and reread and grouped into categories according to their similarities and characteristics.

The first five transcripts were independently coded (M. E. M., J. W. M. v. G.). Codes and themes were compared, after which the coding tree was finalized. A third researcher (R. J. C.) was involved in checking categories and themes. Finally, J. W. M. v. G. analyzed the remaining transcripts. Four reflexivity meetings involving at least five members of the research team were held to explore the diversity of patients' perspectives. During these meetings, codes and themes were revised to make them more comprehensive and clearer. Findings were presented and discussed to increase the richness of data and reduce researcher bias.^23^ The complete data analysis was conducted in Dutch. The English translations of themes, categories, and quotes were crosschecked and adjusted in a separate meeting with native English speakers.

Demographic data of patients were entered into Statistical Package for the Social Sciences version 21. Categorical data were summarised using frequencies and percentages, and continuous data were analyzed using descriptive statistics in case of not normally distributed data.

Four quality criteria for trustworthiness were incorporated into the study design.^24^ Credibility was improved using independent open coding by two researchers and a third researcher involved. Dependability was ensured using member‐checking. Participants received the transcript of their interview and were invited to check the transcript.^25^ To ensure confirmability, probing and prompting questions were used to provide a deeper understanding of participants' answers. To enhance the transferability of the data, outcomes were repeatedly discussed within the research team. All members were trained in qualitative research; six research team members had previous experience with conducting qualitative research (J. M. v. D. /S. W./J. W. M. v. G./M. E. M./M. v. d. S./M. v. d. L.).

## 3 RESULTS

Nineteen interviews were conducted between May 2023 and July 2023. During two interviews, a relative was present to support the participant. A total of 80% (*n* = 15) of participants suffered from multiple chronic conditions; all participants received treatment from at least two different allied healthcare professionals during hospital admission. At the moment of hospital discharge, participants had the following CFS: nonfrail (32%), mild‐to‐moderately frail (58%) and severely frail (10%). Interviews had a median duration of 35 min (interquartile range [IQR]: 16). Participants had a median age of 55 years (IQR: 26), and 63% (*n* = 12) were female (Table 1). Of all participants, 79% (*n* = 15) were discharged home after hospitalization. All participants received their transcript for a member check, and 10% of the participants provided us with feedback. This feedback did not influence our results. Of all the approached participants, two declined to participate in this research due to not having the time to perform an interview.

**Table 1:** Participant characteristics.

Demographic information	Value
Age, median (IQR)	5 (26)
Female, *n* (%)	12 (63)
Department, *n* (%)	
ICU	5 (26)
Trauma surgery	4 (21)
Oncologic surgery	4 (21)
Internal medicine	6 (32)
Admission, *n* (%)	
Acute	5 (26)
Planned	14 (74)
Hospital, *n* (%)	
Academic	14 (74)
General	5 (26)
CFS, after discharge, *n*, (%)	
Nonfrail (1–4)	6 (32)
Mild to moderately frail (5–6)	11 (58)
Severely frail (7–9)	2 (10)
Length of stay, days, median (IQR)	15 (27)
Length of ICU stay, days median (IQR)	16 (17)
Time since hospital discharge in months, median (IQR)	7 (9)
Discharge destination, *n* (%)	
Home	15 (79)
Rehabilitation centre	3 (16)
Geriatric rehabilitation centre	1 (5)

Abbreviations: CFS, clinical frailty scale; ICU, intensive care unit, IQR, interquartile range; *n*, number.

We identified three themes from the data: ‘Allied healthcare support during transition’, ‘Patient and family involvement’ and ‘Information recall and processing’.

### 3.1 Theme 1: Allied healthcare support during transition

In this theme, participants shared their experiences with allied health professionals who supported them in reaching their rehabilitation goals during the different recovery phases: in‐hospital and after hospital discharge. The following three subthemes were identified:

#### 3.1.1 Received support from allied health professionals before discharge

This subtheme relates to participants' perceptions of how allied health professionals collaborated to support them in the in‐hospital phase. During their hospital stay, patients perceived that all (allied) health professionals worked cohesively towards achieving the same goals.

I don't know how they communicate, but I never thought: the physician says one thing and the physical therapist says something else. I never felt as if everyone wanted to go in a different direction [with their therapy]. (Patient 1, 73 years old)

Furthermore, patients appreciated the continuity of allied health professionals. Being treated by the same therapists was reassuring, as patients knew what to expect and felt safe. However, participants felt that often, professionals acted as if they were not communicating with each other. Indeed, some questions were repeatedly asked, for example, questions related to their home situation—which patients perceived as tiring. Nevertheless, participants sometimes noticed that information was exchanged between healthcare professionals, as illustrated by the following quote of a patient whose physical therapy treatments were continued by the nursing staff during evenings or weekends.

Sometimes the nurse fulfilled the role of the physical therapist and sometimes it was the other way around. I didn't have any desire to distinguish between the two. (Patient 3, 83 years old)

Experiencing such continuity of care resulting from collaboration between professionals was highly valued by the participants. Hence, it was satisfying for patients to work towards rehabilitation goals. Participants further experienced the role of allied health professionals during hospital admission as supportive. Patients greatly appreciated the support provided by their physical therapists, as physical therapy allowed them to actively contribute to their own recovery.

The physical therapist was my last resort to get better. My only goal was to get back to my old self, and my physical therapist helped me a lot with that and got me through it. (Patient 5, 34 years old)

The physical therapist has a real impact on how you approach every day and how you recover. Yeah. And that's not the impression I get [of the approach] of the doctor. (Patient 11, 56 years old)

Participants also described experiences with dieticians and speech‐ and language therapists who, according to them, played more of an advisory role in promoting recovery than physical therapists due to a lower frequency of visits. The fact that dieticians were less frequently seen in patients' rooms than physical therapists sometimes resulted in dissatisfaction and negative experiences with dietary restrictions.

It took a while for the dietician and me to be on the same page, but eventually I got the tips I needed. (Patient 2, 60 years old)

Finally, participants noticed that an allied health professional's visit was often planned in consultation with a nurse to ensure it would not coincide with the patients' other appointments during the day. Patients perceived this to be positive because it ensured they were rested and had enough energy during the physical therapy appointment.

#### 3.1.2 Received support from allied health professionals after hospital discharge

This subtheme relates to the phase after hospital discharge. For various reasons, participants generally experienced a smooth hospital‐to‐home transition and were satisfied with the allied healthcare provided after discharge. After hospital discharge, patients usually experience difficulties in physical functioning. So, getting in touch with an allied healthcare provider during this phase was essential as they provided patients with the support they needed. This support was experienced if the professionals discussed the rehabilitation goals with the patients. Patients preferred making an appointment with a primary care professional they already knew and trusted, as being treated by a familiar professional was more reassuring than being treated by an unknown person, even if that person was more trained or specialized in the complaint or disease experienced by the patient.

I was pleased that I [already] knew the physical therapist, she knew how my body works and of earlier things [medical history]. I found that to be very positive. (Patient 5, 34 years old)

I already talked about it [with the physical therapist] at the hospital: ‘just send everything to my physical therapist at home, he knows me, then I will continue it [the treatment] with him’. (Patient 2, 60 years old)

Second, participants found the time between hospital discharge and the start of follow‐up care in the primary care setting short enough, which was satisfying. They felt that care provision and recovery were not interrupted. Sometimes, patients appreciated having a few days to land at home, after which they had their first appointment with their allied health professional.

It was nice to be able to land first, at home, to find out: what am I able to do? After that, they [the physical therapist] came and that was fine. (Patient 4, 31 years old)

Participants also addressed negative experiences with allied healthcare after discharge. For example, the referral letter from the hospital to the primary care professionals consisted of only one‐way communication, meaning that the hospital and primary allied healthcare providers barely had contact with each other.

Really, not one **** is being done [with the referral letter from the hospital], they open it and just continue with their own program anyway. (Patient 12, 44 years)

Patients regretted that they had to tell their stories repeatedly to various professionals. It was suggested that physical therapists involved in the aftercare could share information with other allied health professionals involved. According to the patients, poor communication between the hospital and primary care professionals might have delayed their recovery and increased the burden on relatives who supported them.

My [hospital] physical therapist hadn't read the letter of my primary care [physical] therapist. […] I had told him: ‘I'm going to hospital next week’ and [he said] I'm going to send them an update. But that didn't seem to happen. So, I very often found that… they had missed each other. (Patient 11, 56 years old)

Finally, the long waiting lists for treatment from professionals of certain allied health disciplines in primary care made some patients feel that they were offered care too lateafter they had already found their own solutions.

By the time I could go to therapy, I had already found my groove. (Patient 8, 43 years old)

#### 3.1.3 Relying on oneself

This subtheme relates to the phase after hospital discharge. During this phase, patients indicated to have experienced fear, isolation, and a sense of being a burden to their relatives, for whom the situation felt emotionally and physically heavy. Patients did not want to rely entirely on the help of family members because they were also busy with their daily routines. Therefore, achieving independence was a priority, but patients often missed guidance from allied health professionals on how to recover faster and more efficiently.

It was mainly that I couldn't find my way towards recovery and felt no one was helping. (Patient 9, 76 years old)

I really missed more direct guidance, being taken by the hand to re‐enter the real world. (Participant 12, 44 years old)

Furthermore, participants experienced barriers to contacting their hospital carers when they had specific questions or felt unwell. They sometimes believed that their problems were not severe enough to warrant calling health professionals at the hospital and feared disturbing them. Therefore, some patients decided to accept the presence of their problems and unanswered questions, sometimes for weeks, until their scheduled appointment with the medical specialist at the hospital. Patients who sought assistance and telephonic communication with their healthcare providers at the hospital sometimes encountered difficulties reaching them, as the administrative personnel answering the phone rebuffed them. This resulted in a feeling of not being taken seriously.

I was even ready to go to the emergency room. Because there [at the emergency room] I have an entry—they will examine me again. And if I phone them, there is no answer at all. (Patient 5, 34 years old)

Finally, participants mentioned some needs and suggestions which they thought would have been beneficial to them. Receiving a follow‐up call from a health professional within 6 weeks after discharge or having the availability of a telephone number to call in the event of an emergency would be valued. Thereby, they would have valued contact with peers and have a contact person who is available to guide patients throughout the whole transition process.

### 3.2 Theme 2: Patient and family involvement

Participants explained in which way they were involved in the decision‐making surrounding the discharge process. The following quote illustrates the experience of a patient who was not feeling ready to be discharged and requested to stay an extra night in the hospital, which was granted. This patient indicated that he/she felt heard, which, in turn, was reassuring and empowering.

I felt I was entitled to one more night [in the hospital], and I ended up getting it, and that was nice. (Patient 3, 83 years old)

The moment of discharge seemed sudden and unexpected to some of the participants. In those instances, they felt that they were not ready for discharge, both emotionally and physically, leading to feelings of insecurity.

Well, it was what we wanted, but it all went so fast and there was no build‐up or working towards discharge. I got a stack of papers and they wished me luck and that was that: There's the door. (Patient 16, 50 years old)

On the contrary, some patients experienced a sudden discharge as a relief since they wanted to go home as soon as possible.

Furthermore, patients and family members highly value the involvement of relatives in the discharge process. First, family members often have a more realistic image of the home environment. They know and can describe the patient's situation and status before hospital admission, which is crucial information for allied health professionals to consider when designing follow‐up treatment recommendations. Second, they can assist their loved ones in arranging aftercare, such as acquiring walking aids or procuring medication. Patients emphasised that organising aftercare was only doable with the help of their families.

My family was really involved, because they would need to help (after discharge) and that was quite nice. (Patient 4, 31 years old)

### 3.3 Theme 3: Information recall and processing

This last theme pertains to the quantity, clarity, and timing of received information as essential aspects to be considered by allied health professionals to facilitate patient information recall and processing. We identified the following subthemes:

#### 3.3.1 Timing of receiving information and presence of relatives

Information provided on the day of hospital discharge generally did not stick with the patients because their focus was on going home, which required most of their energy and attention. Recalling information provided at this moment is, therefore, challenging. In addition, patients indicated that this information was not always given in the presence of family members, who could have helped them recall it. Not remembering information resulted in feeling lost and not knowing how to self‐manage their recovery after arriving home.

I was left, with a lot of questions [when I arrived home]. (Patient 14, 70 years old)

#### 3.3.2 Quantity and clarity of information

Participants mentioned negative experiences with the amount of information they received, which was sometimes overwhelming or incomplete. The following quote relates to a patient who felt lost in a labyrinth of information and unable to process that amount of information.

Yes, I quickly found it all very confusing, you have to arrange everything by yourself. You receive discharge papers for the physical therapist, occupational therapist, but you have to make all arrangements by yourself. (Patient 17, 46 years old)

On top of not receiving the right amount of information, participants felt that the information provided needed to be clarified and adjusted to their level of comprehension. As depicted in this quote, the use of medical or Latin terms by healthcare professionals was negatively perceived:

We are not medically trained, and they use lots of medical terms, so it [the information] should be more in layman's terms. (Patient 13, 18 years old)

Finally, participants explained that healthcare providers did not check whether the provided information was understood. Again, this led to being left with questions and not knowing how to self‐manage at home.

Afterwards [at home], more questions arose because I did have delirium. I was given some information about it, but I kept wondering what you can do about it at home. (Patient 18, 52 years old)

Finally, participants mentioned they would have benefited from clear information about the disease and expected recovery time. They wished to have a checklist that would assist them in arranging aftercare, would clarify what type of aftercare was applicable to them, and how they could arrange it. Additional quotes to support the results section are provided in Appendix S3.

## 4 DISCUSSION

This study aimed to gain insight into patients' experiences, perceptions, and needs regarding hospital‐to‐home transition, focusing on allied healthcare as a first step towards the development of a transitional integrated allied healthcare pathway for patients with complex care needs after hospital admission. The data analysis revealed three themes: ‘Allied healthcare support during transition’, ‘Patient and family involvement’ and ‘Information recall and processing’.

The first theme—*Allied healthcare support during transition*—emphasises the importance of allied health professionals supporting patients in reaching rehabilitation goals. On the one hand, this study depicts the positive experiences of participants who felt they could rely on allied health professionals who tailored therapy to their needs, ensured continuity of care and established a trusting relationship with the participants to reassure them and enhance their recovery. Providing tailored therapy, ensuring continuity of care, and establishing a trusting relationship with patients are essential goals to be reached by healthcare professionals as they can improve patient satisfaction, provide a sense of security, and increase adherence to prescribed treatments.^26^ On the other hand, our study participants encountered negative experiences regarding communication with allied health professionals at the hospital after being discharged home and poor communication between hospital‐based and primary care‐based allied health professionals. These communication issues resulted in patients not receiving the support they needed.^11^ Poor communication postdischarge has been found to lead to frustration, delayed follow‐up, and potentially delayed recovery.^27^ Effective communication with patients is still not optimal in many settings worldwide.^28^, ^29^, ^30^ Therefore, there is a global need to improve the process for transferring information to patients and their primary healthcare providers. Many studies have attempted to address communication issues through various transitional care interventions.^28^, ^29^, ^30^ However, these interventions were usually targeting medical needs, nurse‐led and primarily involved communication interventions such as follow‐up calls. Patients with complex care needs after hospitalization typically require a more comprehensive approach involving several professionals from allied health disciplines, providing integrated care reflecting a more holistic approach. However, such a patient‐centred model of care involving hospital and primary care professionals has yet to be developed. To achieve this, recent studies have considered using innovative digital applications, such as mobile apps, which are promising solutions that can enable the timely provision of information and support and empower patients to reach their goals.^31^ More research is needed to determine how to integrate these solutions at an organizational and system level.^32^

The second theme—*Patient and family involvement*—emphasises the importance of involving patients and relatives in the discharge process. It highlights that involving patients and relatives in discharge planning can empower patients. In contrast, sudden discharge leads to feelings of uncertainty and insecurity. These findings align with previous studies that found that the information concerning discharge was often provided unexpectedly and without considering patients' preferences.^11^, ^33^ A qualitative study on transitional care decision‐making from the perspective of older people distinguished two subgroups of patients. The first subgroup did not want to be involved in making decisions related to their care and preferred relying on their formal care providers, whereas the second subgroup felt they were given no choice and that the transitional care decisions were forced upon them.^34^ Kraun et al., therefore, pointed out that patients have different preferences that must be considered.^34^ These findings suggest that listening to patient preferences and involving family members is crucial to providing person‐centred care. Future research should focus on incorporating these factors and clarifying the role of allied health professionals to enhance the integration and continuation of care during the hospital‐to‐home transition.

The third theme—*Information recall and processing*—underscores the need for personalized information that is timely and effectively provided and in the presence of family members if possible. Our findings showed that processing or recalling information is often challenging for several reasons. Depending on the patient, information can be overwhelming, and using complicated medical terms can lead to negative feelings such as fear. Each patient has specific needs, and professionals must continually assess them to ensure that they provide comprehensible information at the right moment. Our findings confirmed the findings of previous studies that also emphasised that access to information is an essential element in the transitional decision‐making process and that the type of information needed, how it is delivered, and when should be tailored to patients' individual preferences, which is consistent with our results.^34^, ^35^, ^36^ Furthermore, it is well established that patient recall is generally very low. Kessels and collegues found that, in general, up to 80% of the information given by healthcare professionals was forgotten, and this is confirmed by more recent studies, meaning that finding adequate interventions to address this continues to be a challenge.^37^, ^38^ In our study, patients mentioned that the involvement of families when providing information would help them recall discharge instructions. Therefore, any future integrated care pathway should involve relatives during information delivery, although research is needed to determine precisely how this should be done.^33^

### 4.1 Strengths and limitations

In this study, we included patients from academic and general hospitals with diverse reasons for hospital admission and discharge destinations. Consequently, our findings regarding allied healthcare needs after hospitalization may apply to a broad population. This wide variation in participants may also affect the validity of our results, and it is important for clinicians to consider the needs and desires of the individual patient. While several qualitative studies among hospitalized patients investigating transitional experiences are available, this is the first study focusing on allied healthcare. Furthermore, this study fills a knowledge gap because many studies on transitional care interventions focus on nursing perspectives, and the methodological rigour we applied contributes to the transparency of our results. We did make use of a member check and involved multiple researchers in the analysis process; we could have used more strategies to enhance the transferability of the data.

However, there are also limitations to our study. First, we included several vulnerable participants for whom it was sometimes difficult to explain and share experiences due to cognitive and emotional problems related to their hospitalization. Consequently, it was sometimes difficult for patients to specifically recall experiences regarding some critical elements of the discharge process. This could have influenced the accuracy and volume of the memories of the experiences. We aimed to vary the time between hospital discharge and interview within our study population in order for the recall bias not to be detrimental to our research. Second, patients' knowledge of J. W. M. v. G.'s association with AUMC might have led those patients who had been admitted to this hospital to provide desirable responses. However, there was no prior relationship between any of the participants and the principal researcher. Third, bias may have occurred due to recruitment strategies. This may have resulted in the selection of very satisfied or very dissatisfied participants. Participants who were recruited via the outpatient clinic or other research projects within the AUMC had outpatient clinic visits before or after hospital admission. Lastly, most participant experiences with allied healthcare concerned physical therapy. The participants shared fewer interactions with dieticians, occupational therapists and speech‐ and language therapists, while it is possible that they could have benefited from interventions provided by these professionals. This should be considered when designing a transmural allied health pathway. Nevertheless, we believe the current selection of participants and their experiences are representative of the study population.

## 5 CONCLUSION

This study clarifies the experiences, perceptions, and needs of patients with allied healthcare needs in their hospital‐to‐home transition. Our findings show that hospital and primary care allied health professionals need to continuously collaborate and communicate with each other to provide patients and their families with the personalized support they need. Particular attention should be given to how, when, and what type of information is provided to patients and their relatives to facilitate self‐management and active involvement in their recovery process. These findings should be considered when (re)designing an integrated allied health pathway aimed at facilitating communication and adequate information exchange between professionals, patients, and relatives.

## AUTHOR CONTRIBUTIONS

**Juul W. M. van Grootel**: Conceptualization; writing—original draft; methodology; formal analysis; project administration; software; writing—review and editing; resources. **Romain J. Collet**: Conceptualization; writing—review and editing; formal analysis; methodology. **Mel E. Major**: Conceptualization; methodology; writing—review and editing; formal analysis. **Suzanne Wiertsema**: Conceptualization; methodology; writing—review and editing; formal analysis. **Hanneke van Dongen**: Conceptualization; writing—review and editing; formal analysis; methodology. **Marike van der Leeden**: Conceptualization; writing—review and editing; formal analysis; methodology. **Edwin Geleijn**: Conceptualization; writing—review and editing; methodology; formal analysis. **Raymond Ostelo**: Conceptualization; writing—review and editing; formal analysis; methodology. **Marike van der Schaaf**: Conceptualization; methodology; writing—review and editing; formal analysis.

## CONFLICT OF INTEREST STATEMENT

The authors declare no conflict of interest.

## ETHICS STATEMENT

The medical ethics committee of the Amsterdam University Medical Centers provided a waiver for this study (METC 2023.0119). All patients participating in this research provided written informed consent.

## Supporting information

Supporting information.

Supporting information.

## Data Availability

The data that support the findings of this study are available on request from the corresponding author. The data that support the findings of this study are openly available in DataVerseNL at https://doi.org/10.34894/PCG9HO.
