# Supplementary material for: Engaging patients in designing a transmural allied health pathway: A qualitative exploration of hospital‐to‐home transitions
Source: Health Expect. 2024 Mar 15;27(2):e13996. doi: 10.1111/hex.13996 (PMC10943249; doi:10.1111/hex.13996)
Supplement: Supplementary file 1 — Supporting information. [file HEX-27-e13996-s001.docx]

**Appendix 1: The Dutch healthcare system**

*Principles of the Dutch health care system*

The current Dutch health care system can best be explained by looking at a number of recent changes. In 2006 the new Health Insurance Act entered into force, under which all residents of the Netherlands are entitled to a comprehensive basic health insurance package. This act is implemented by private, competitive health insurers and health care providers. It should be noted that virtually all health insurance companies in the Netherlands are not-for-profit cooperatives that allocate any profits they make to the reserves they are required to maintain or return them in the form of lower premiums.^1^

The Dutch healthcare system is divided into three compartments:

- Long-term care for chronic conditions.
- Basic and essential medical care, from GP visits to short-term hospital stays and specialist appointments or procedures.
- Supplementary care (e.g. dental care, allied healthcare, cosmetic procedures).

Long-term care, including disability costs like wheelchairs, is covered by mandatory state insurance.

All regular (short-term) medical treatment is paid for by mandatory private health insurance. Supplementary care may be covered under health insurance, depending on the policy, or be paid for out of pocket.

*Doctors & GPs*

Doctors and general practitioners are your first point of contact for healthcare in the Netherlands, as they provide referrals to all specialists and, if necessary, to a hospital. Registering with a local practice is one of the first things you need to do.

*Hospitals*

Hospitals in the Netherlands fall into three categories:

- Academic, for specialist care and research.
- Teaching, for training healthcare practitioners.
- General, for less specialized care.

They all provide a high level of care, but they are moving to greater specialization in different areas.


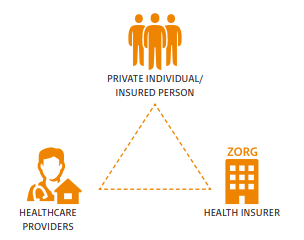


Figure 1 The Dutch healthcare system, adopted from Ministry of Public Health ^1^

In the Netherlands, hospital payment rates are primarily established through negotiations between health insurers and hospitals, focusing on prices, quality, and service volumes. The key payment method is the Diagnosis-Treatment Combination (DTC) system. A DTC serves as a record containing information about the diagnosis, treatment, and costs associated with both inpatient and outpatient services provided by hospitals. Fixed amounts are charged for each episode of care, with individual prices assigned to each DTC.^2^

This system affords a level of adaptability and tailoring in healthcare payment agreements, as the dynamics of negotiations between insurers and hospitals play a pivotal role in determining the financial aspects of healthcare services. It reflects a prospective payment model where costs are predetermined for specific care episodes, contributing to a degree of financial predictability in healthcare expenditures.

In response to the financial crisis in 2013, the government introduced a cost-saving initiative known as "the right care at the right place". This strategy sought to mitigate expenses by substituting relatively costly in-hospital care with care provided in primary care.^3,4^

1. Ministry of Public Health, Welfare and Sport, Dutch Healthcare in the Netherlands (2016) accessed on 11-01-2024 via <https://english.zorginstituutnederland.nl/about-us/publications/publications/2016/01/31/healthcare-in-the-netherlands>
2. Tikkanen R. OR, Mossialos E., Djordjevic A., Wharton G. A. . International Health Care System Profiles: Netherlands. In: Fund TC, editor. 2020.
3. Jeurissen P, Maarse H. European Observatory Health Policy Series. The market reform in Dutch health care: Results, lessons and prospects. Copenhagen (Denmark): European Observatory on Health Systems and Policies © World Health Organization 2021 (acting as the host organization for, and secretariat of, the European Observatory on Health Systems and Policies). 2021.
4. VWS. The right care in the right place 2018 [Care in the Right Place Taskforce]. Available from: https://www.dejuistezorgopdejuisteplek.nl/.uc/fcef77d2b01028d5c0000bd7ca7026baaac90942d76c900/The%20right%20care%20in%20the%20right%20place_report%20taskforce.pdf.
