# Supplementary material for: Engaging patients in designing a transmural allied health pathway: A qualitative exploration of hospital‐to‐home transitions
Source: Health Expect. 2024 Mar 15;27(2):e13996. doi: 10.1111/hex.13996 (PMC10943249; doi:10.1111/hex.13996)
Supplement: Supplementary file 2 — Supporting information. [file HEX-27-e13996-s002.docx]

Appendix 2: Interview guide:

**English translation of the interview guide:**

**During hospitalization: experiences with allied healthcare**

- How long has it been since you were hospitalized, and can you tell us about the reasons for your admission and how long you were hospitalized?
- We would like to know more about the allied healthcare you received during hospitalization. What kind of care did you receive in the hospital? Think about the care you received from the physical therapist, occupational therapist, dietician or speech and language therapist. This is what we call allied healthcare.
- Looking back now at that experienced allied care, can you indicate what you experienced positively about allied healthcare and what you may have found less positive?
  - For example, ask: did the allied healthcare treatment fulfill your needs?
- Did you miss any care or information regarding nutrition and exercise?
  - If yes; In your opinion, did missing this care result in any adverse effects on your recovery?
  - If not; In your opinion, did this care contribute positively to your recovery?

Summary with carer/family member: is this also how you experienced it?

**During hospitalization: towards hospital discharge**

- You probably had to prepare yourself for discharge when you were in the hospital. How did you experience discharge from the hospital? Consider the time to discharge, discharge information, and communication you received about your discharge. Was discharge and discharge destination discussed with you, and if so, how?
- Was your family involved in the preparations for your discharge?
- How was hospital discharge organized for you? Where did you go? Did you go home or to a nursing home or rehabilitation center?
- What follow-up was organized for you from the hospital (think about medication, district nursing, etc.)
- Who discussed the discharge plan with you? Which healthcare providers were involved?
- What role did you play in planning your discharge?

Summary with carer/family member: is this also what you experienced? To what extent were the discharge plans discussed with you? Think also about who discussed discharge plans with you. Was this face-to-face or by phone? Was this discussed with you promptly?

**After discharge from the hospital**

- We would like to know how things went with you after discharge to get a good picture of your transition from hospital to home. How did you feel after discharge from the hospital (for example, what about your physical, cognitive, and emotional status)?
- From whom (which professionals) did you receive follow-up care after discharge from the hospital? How did you get in touch with these professionals? (Did you arrange it yourself, or via your GP, or did the hospital assist you?) Was there a transfer of your (medical) information from the hospital to the professionals who provided you with follow-up care? Did the hospital arrange this information transfer, and was this done promptly?
- Which other people did you receive help from? Perhaps the physiotherapist came to your home, the occupational therapist, dietician, or speech and language therapist? Did this happen directly after discharge from the hospital, or was there consciously/or unconsciously a time in between?

Summary with carer/family member: is this also what you experienced?

**Collaboration of allied health professionals**

- As mentioned at the beginning, the intervention of different healthcare professionals with different specializations can help you recover better. How did you experience the collaboration between these healthcare professionals (physical therapist, occupational therapist, dietician, speech and language therapist, general practitioner) after you were discharged? Was there a difference between hospital professionals and primary care professionals?
- Were healthcare providers inside and outside the hospital in contact with each other?
- Did you notice if these professionals were consulting each other (in making plans for your recovery)? What did you notice exactly? And if not, is this something you felt was missing?

Summary with carer/family member: is this also how you experienced it?

**Areas for improvement**

- What could have made your transition from hospital to home better? And why?
- What could your allied health providers have done better? What did you miss in your care? What could have been done differently in the hospital? What could have been done differently after returning home?

**Additional questions**

- Suppose you are hospitalized again. What would you pay attention to right away?
- Could you describe what the ideal situation would look like?
- What did you really miss in the transition from hospital to home?
- How would you feel about using digital communication tools to facilitate this transition?
- Based on your overall transition experience, what else did you need to make it more optimal?

Appendix 3: Additional quotes

| **Theme** | **Subtheme** | **Quote** |
| --- | --- | --- |
| Allied healthcare support during transition | Received support from allied health professionals before discharge | *“I only have positive things to say about physical therapy, as soon as you start moving little by little, you feel better. You feel your strength returning.”*  (Patient 2, 60 years old) |
|  | Experiences of allied healthcare after hospital discharge | *“[A visit from a ] Dietician after discharge, I did not feel the need for that, as I had been admitted to the hospital for a very long time so at some point I knew everything.”*  (Patient 5, 34 years old) |
|  | Relying on oneself | *“You just want clear-cut tips. And that they clearly state: I will be doing this now, so you will receive that. That is what you want, isn’t it? You want to know, when will I arrive at the next spot [at the horizon]? And not just wandering around all the time.”*  (Patient 2, 60 years old) |
| Patient and family involvement |  | *“Well, it felt every time, very much like it was up to me. It was like, if you want to go home, you can go home. And then that was agreed on the basis of me being able to get out of bed.”*  (Patient 11, 56 years old) |
|  |  | *“We were lucky enough that my wife could sleep in the room with me, so she was very much involved with everything that happened to me – she very much had a say in this, that was very nice.”*  (Patient 6, 59 years old) |
| Information recall and processing | Timing of providing information and presence of relatives | *“I was told about the possibility to go to the rehabilitation center at the moment a friend of mine visted me. She ended up taking over the conversation as it was too much information for me. Someone can bring it real nicely, but I just couldn’t process it at that time.”*  (Patient 12, 44 years old). |
|  | Quantity and clarity of information | *“Yes, I need to think hard but I can really not be sure about all the information that I was given.”*  (Patient 6, 59 years old). |
